# ABCC7/CFTR Expression Is Associated with the Clinical Course of Ulcerative Colitis Patients

**DOI:** 10.1155/2021/5536563

**Published:** 2021-08-31

**Authors:** Marco A. Villeda-Ramírez, Daniela Meza-Guillen, Rafael Barreto-Zúñiga, Jesús K. Yamamoto-Furusho

**Affiliations:** ^1^Inflammatory Bowel Disease Clinic, Department of Gastroenterology, Instituto Nacional de Ciencias Médicas y Nutrición Salvador Zubirán, Mexico; ^2^Postgraduate in Biological Sciences, Universidad Nacional Autonoma de México, Mexico; ^3^Department of Endoscopy, Instituto Nacional de Ciencias Médicas y Nutrición Salvador Zubirán, Mexico

## Abstract

Inflammatory bowel disease includes ulcerative colitis (UC) and Crohn's disease (CD) of unknown etiology. The expression of ATP-binding cassette (ABC) family proteins has been associated with drug resistance and development of UC. The cystic fibrosis transmembrane conductance regulator (CFTR) or also known as ABCC7 is involved in the inflammatory chronic response. The aim of this study was to evaluate the role of ABCC7/CFTR in UC patients and normal controls without inflammation. This is an exploratory, observational, and cross-sectional study that included a total of 62 patients with UC and normal controls. Gene expression of CFTR was measured by RT-PCR, and protein expression of CFTR was determined by western blot analysis. We found a significant downregulation of the CFTR gene expression in patients with active UC compared to normal controls without inflammation (*P* < 0.004); even the gene expression of CFTR was decreased in remission UC patients compared to normal controls without inflammation (*P* = 0.04). The CFTR gene expression was associated with the clinical course of UC and the protein expression of CFTR was decreased in active UC patients compared to normal controls without inflammation suggesting that this molecule might play a role in the inflammation in UC patients.

## 1. Introduction

Ulcerative colitis (UC) is a chronic inflammatory condition of the colon that affects only the colonic mucosa of unknown etiology. The clinical symptoms include rectal bleeding, chronic diarrhea, and abdominal pain [1, 2]. It has been proposed that innate and adaptive immunity is involved in the inflammatory response; specifically in UC, there is an increase in the production of proinflammatory interleukins (IL) such as IL-1, IL-6, and IL-8 as a result of activation of the transcriptional nuclear factor *κ*B (NF-*κ*B) [3–5]. Recently, genes that encode for a family of ABC transmembranal multidrug-resistant proteins (ABC (ATP-binding cassette) family) have been associated with medical response and clinical course of disease [6]. The ABC family is composed by 48 transmembranal proteins grouped into 7 subfamilies (ABCCA-ABCCG) [7, 8]. The ABCC member 7 (ABCC7), also known cystic fibrosis transmembrane conductance regulator (CFTR), is an ionic channel that actively participates in the regulation of elimination and absorption of chloride ion in different tissues including the gastrointestinal tract [9, 10]. The presence of mutation in the ABCC7/CFTR gene has been associated with cystic fibrosis (CF) which is a prevalent disease in white populations; dysfunction and changes in gene expression have been implicated in other disease development, including chronic inflammation [11, 12]. In patients with CF, an increase of fecal calprotectin has been observed, the same as what happens in inflammatory bowel disease (IBD) [13–15]. The ABCC7/CFTR knockout mouse model developed intestinal inflammation suggesting a role of this gene in gut inflammation [12, 16]. The levels of ABCC7/CFTR expression have been involved in modulating the NF-*κ*B activity and the production of IL-8 and IL-6 in lung cell lines [17–19]. The aim of this study was to determinate the ABCC7/CFTR gene and protein expression in UC patients and its association with clinical outcomes.

## 2. Materials and Methods

This is an exploratory, observational, and cross-sectional study that included 62 individuals who were consecutively recruited and divided in 3 groups: active UC (*n* = 20), remission UC (*n* = 21), and normal controls without inflammation (*n* = 21). All patients belonged to the Inflammatory Bowel Disease Clinic at the Instituto Nacional de Ciencias Medicas y Nutrición Salvador Zubirán. All patients had a definitive diagnosis of UC confirmed by histopathology. All demographical and clinical variables were collected from interview and medical records such as age at diagnosis; gender; type of medical treatment (5-aminosalicylates, steroids, thiopurines, and biologic therapy); disease extension or location according to Montreal classification; the presence of extraintestinal manifestations such as articular affection, ankylosing spondylitis, sacroiliitis, primary sclerosing cholangitis, pyoderma gangrenosum, erythema nodosum, or uveitis; and clinical course classified as initially active and then prolonged remission (first episode with activity and then long-term remission for more than 5 years), intermittent activity (>2 relapses per year), and chronic continual activity (persistent activity despite medical conventional therapy). The clinical and endoscopic activity was determined by Total Mayo Score (TMS) and Mayo endoscopic score (MES) and histological activity by Riley score [20, 21], and a novel integral disease activity index for UC or Yamamoto-Furusho index was also used that considers clinical, biochemical, endoscopic, and histologic parameters [22]. Active disease was defined by the presence of more than 2 points in the TMS, MES ≥ 1, and active disease by Riley score was the presence of the following 6 variables: (1) acute inflammatory infiltrate (polymorphonuclear cells at the lamina propria), (2) chronic inflammatory infiltrate (lymphocytic infiltrate at the lamina propria), (3) cryptitis (abscesses), (4) loss of epithelial integrity, (5) mucin depletion, and (6) irregularities in the architecture of the crypts. Finally, active disease by the Yamamoto-Furusho index was defined by ≥4 points; this index is divided into (1) mild activity (4 to 6 points), (2) moderate activity (7 to 12 points), and (3) severe activity (13 to 18 points) [22]. Remission UC was defined by the presence of TMS ≤ 2 points, MES = 0 points, and Riley score by the absence of acute inflammatory infiltrate (polymorphonuclear cells at the lamina propria), (2) chronic inflammatory infiltrate (lymphocytic infiltrate at the lamina propria), (3) cryptitis (abscesses), (4) loss of epithelial integrity, (5) mucin depletion, and (6) irregularities in the architecture of the crypts as well as Yamamoto-Furusho index ≤ 3 points. All patients in remission should fulfill the abovementioned index criteria. The control group consisted of colonic biopsies of individuals without colonic inflammation defined by the lack of clinical, endoscopic, and histological data on intestinal inflammation or systemic disease (cancer, autoimmune disease, diverticular disease, medicated colitis, postradiation colitis, infectious colitis, and ischemic colitis). The absence of intestinal inflammation was verified histologically in the normal control group.

### 2.1. Tissue Samples and Gene Expression Analysis

All colonic biopsies were taken by colonoscopy with written informed consent of all the patients that participate in the study, immediately placed in RNAlater (Ambion, Austin, TX, USA) and stored at -70°C until processing. Total RNA extraction from colonic biopsies was made using RNA extraction kit (High Pure RNA Tissue Kit, Roche). The biological sample was mixed by homogenizer Polytron® LD1300 for 1 minute; the RNA was extracted by commercial kit for RNA extraction High Pure RNA Tissue kit (Roche®) after the incubation with DNase for 20 minutes and was diluted in 50 *μ*l of elution buffer and stored at -70°C. The integrity of RNAm was evaluated by electrophoresis in an agarose gel at 1% with ethidium bromide; it was then visualized using an UVP dual-intensity transilluminator.

The cDNA was synthetized through reverse transcription with the cDNA Transcription Synthesis Kit (Roche®) under preincubation at 25°C × 10 minutes, incubation at 55°C × 30 minutes, followed by denaturalization at 85°C × 5 minutes in the GeneAmp PCR System Thermocycler (PerkinElmer®). The RNAm expression of ABCC7/CFTR was measured by real-time polymerase chain reaction, and *β*-actin was used as reference gene. The IL-6 gene expression was used as inflammation marker as previously reported and demonstrated good correlation with histologic activity. For determining the ABCC7/CFTR, IL-6, and *β-*actin expression, we used a set of designed forward and reverse primers (ABCC7/CFTR left: ctgactgtttccatcaagggta, right: gcagtcttcttaagagtcagtttgg; IL-6 left: gatgagtacaaaagtcctgatcca, right: ctgcagccactggttctgt; and *β-*actin left: tccaaatatgagatgcgttgtt, right: tgctatcacctcccctgtgt) and a mix of Taq Man Master with specific probes for human of High Probe Library (Roche®) Real-Time (RT-PCR) was performed in LightCycler 2.0 Roche® thermocycler, under a program of 1 cycle of denaturalization (95°C × 10 minutes), 45 cycles of amplification (95°C × 10 seconds, alignment 60°C × 10 seconds, extension 40°C × 30 seconds), and 1 cycle of cooling (40°C × 30 seconds).

### 2.2. Protein Expression Analysis by Western Blot Analysis

The total protein of colon tissue samples of 4 patients per group (active and remission UC and control group) was extracted with solution of RIPA buffer (Sigma-Aldrich) and cocktail of protease inhibitor (Roche®), quantified by Bradford assay (Bio-Rad, Hercules, CA, USA), and stored at -70C. The protein detection was performed by electrophoresis in SDS-PAGE and then transferred to polyvinylidene difluoride membranes. All blots were blocked with 3% BSA for 60 min at room temperature and incubated overnight at 4 with primary antibodies. The primary antibodies for CFTR/ABCC7 were used in a concentration of 1 : 1000, and *β*-actin was used to normalize the data in a concentration of 1 : 10000. The blots were incubated with anti-rabbit secondary antibodies conjugated with horseradish peroxidase (1 : 3000). Images were analyzed with the ChemiDoc™ XRS+ System Image Lab™ Software (Bio-Rad, Hercules, CA, USA), and the western blot analysis was performed using independent blots.

### 2.3. Statistical Analysis

The statistical analysis was performed using SPSS version 17.0, using Kruskal Wallis nonparametric test, Spearman's correlation, Fisher's exact test, and odds ratio (OR) in order to determine the strength of association. Protein expression statistical analysis was done by using one-way analysis of variance on ranks by Dunn's method for all pairwise multiple comparison procedure (Sigma Stat 11.2 program, Aspire Software International, Leesburg, VA, USA). Data were expressed as median, range, and mean ± standard deviation (SD)/standard error of the mean (SEM). A *P* value < 0.05 was considered as statistically significant.

## 3. Results

### 3.1. Demographic and Clinical Characteristics of UC Patients

A total of 41 patients with UC and 21 normal controls were included in the study. All demographic and clinical characteristics are shown in Table 1.

### 3.2. Gene Expression of CFTR/ABCC7 and IL-6 in Tissue Samples from UC Patients

**Figure 1:**
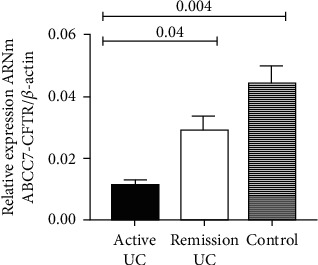
Gene expression of ABCC7/CFTR from colonic biopsies of UC patients. The relative gene expressions of ABC7/CFTR were compared in patients with active and remission UC and the control group. The medians of relative expression in the three groups were compared with Kruskal Wallis and for two groups were analyzed by Mann–Whitney *U* test.

We found a downregulation of the gene expression of CFTR in the active UC group compared to control without inflammation (*P* < 0.004). We also identify that the expression of CFTR was downregulated in the UC remission group compared with control without inflammation (*P* < 0.04) as shown in Figure 1.

**Figure 2:**
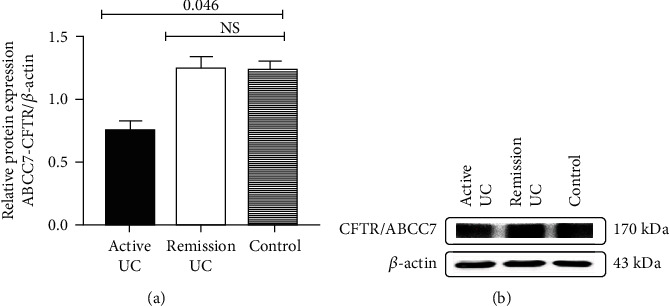
Protein expression of ABCC7/CFTR in colonic biopsies of UC patients.

We observed low protein expression of CFTR/ABCC7 in colonic biopsies of the active UC group compared with the remission UC and normal control groups (*P* = 0.046); no difference was observed in the remission UC group compared with control (Figure 2).

**Figure 3:**
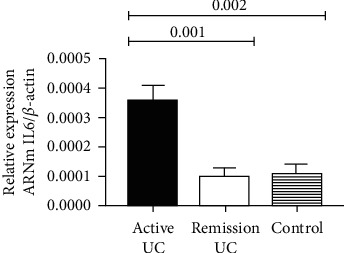
Gene expression of IL-6 from colonic biopsies of UC patients. The relative gene expression of IL-6 was employed as an inflammatory marker and was also compared in patients with active and remission UC and the control group for confirmation of the histological diagnosis. The medians of relative expression in the three groups were compared with Kruskal Wallis *H* and individual groups were analyzed by Mann–Whitney *U* test.

The IL-6 gene expression was measured in colonic biopsies from UC patients in order to determine the presence of inflammation, and it was also correlated with histological activity. The IL-6 gene expression was low in controls without inflammation and UC remission patients compared to the active UC group (*P* < 0.001). The gene expression of IL-6 in the UC remission group and control without inflammation was similar in both groups as shown in Figure 3. No correlation was found between CFTR gene expression and UC patients (*R*^2^ = 0.23, *P* = 0.34) and normal controls (*R*^2^ = 0.11, *P* = 0.68).

The gene expression of ABCC7/CFTR was associated with the clinical course of disease (*P* = 0.005, OR = 21.7, and 95% CI: 3.59–132.0) as shown in Table 2.

The protein expression of ABC7/CFTR was compared in colonic biopsies of patients with active and remission UC and normal control group (Figure 2(a)). The qualitative protein expression is shown in Figure 2(b) and was performed by western blot analysis.

## 4. Discussion

Our study demonstrated a downregulation of the gene and protein expression of CFTR or ABCC7 in patients with active UC compared to UC in remission and normal controls without inflammation. It is important to note that low CFTR gene expression was significantly associated with clinical course characterized of persistent UC.

It is well known that CFTR has been widely studied in cystic fibrosis (CF), and recently, it has grown interest to study its role in other diseases or inflammatory conditions such as in the biliary epithelium and intestine [12, 23–25]. Animal models and in vitro studies have demonstrated the role of CFTR in the intestinal inflammatory process characterized by histological infiltration of immune cells and increase of inflammatory fecal biomarkers [13–16]. Our findings are similar to those reported by Werlin et al. and Lee et al. who found increased levels of fecal calprotectin in children with CF [13, 15]. In vitro studies have demonstrated that a downregulation of CFTR is contributing to chronic inflammation as we found in patients with active UC. This could be explained by findings reported by Wang et al. who reported the effect of mutant ABCC7/CFTR in bronchial epithelial cell line (CFBE cells) during TNF*α* recombinant stimulation; they observed that ABCC7/CFTR mutant increases the activity of transcriptional nuclear factor *κ*B (NF-*κ*B) and production of proinflammatory cytokines compared to those with normal function of CFTR [17].

There were similar findings by Li et al. who found an increase in the NF-*κ*B activity and IL-8 and IL-6 production in human bronchial epithelial cells (16HBE) that had a downregulation of ABCC7/CFTR [18] as well as in patients with human lung adenocarcinoma cell lines [26]. In other cell lines from colorectal carcinoma, Crites et al. reported that a downregulation of ABCC7/CFTR increased the production of IL-6, IL-1*β*, and IL-8 in CACO2 and HT29 cells due to the activation of ERK1/2, MAPK, I*κ*B*α*, and NF-*κ*B pathways [27]. In a knockout animal model of ABCC7/CFTR, they found that the downregulation of CFTR produced inflammation through the increase of NF-*κ*B, TNF*α*, and IL-6 in the mouse intestine and CACO2 cells and cholangiocytes stimulated by lipopolysaccharides from *Escherichia coli* [24, 27, 28]. In another study performed in rats and 16HBE140 cells, it was found that downregulation of CFTR increased the production of IL-8 compared to those that overexpressed CFTR with lack of MAPK/NF-*κ*B activation, and the same finding was also observed in mutant CFTR (DF508) mice and HaCaT cells in which the downregulation of the CFTR expression exhibited inflammation and delayed cutaneous wound healing [29].

Our findings are similar to the abovementioned studies confirming the role of downregulation of CFTR in the development of inflammatory process in patients with active UC.

Nevertheless, it is already known that CFTR interacts with a variety of proteins and regulates its function. Recent studies have demonstrated higher levels of IL-17 in a CFTR knockout mouse model compared to wild-type mouse during infection with *Pseudomonas aeruginosa* resulting in the perpetuation of inflammatory process by neutrophil recruitment suggesting that the absence of CFTR contributes to the inflammation by IL-17 production [30] and which have been also observed in patients with active UC [31].

The CFTR is a channel expressed in the intestine that regulates the efflux of chloride and bicarbonate ions and also participates actively in the pH regulation [9, 10]. A proposed hypothesis explaining the role of CFTR in UC patients is that downregulation of CFTR affects the pH balance in the colon producing a decreased expression of MUC11 and MUC12 contributing to the adherence of bacteria and promoting the colonic inflammation [10, 11, 32], and a decreased gene expression of MUC20 in patients with active UC compared to UC remission and normal controls was reported by our group [ 33].

On the other hand, changes in the intestinal pH and decreased MUC11 and MUC12 expression promote the colonization of phatobiontic microbiota in the intestinal lumen as it has been reported in IBD patients [11, 24, 34]. Phatobiontic microbiota increased the expression, recognition, and activation of TLR4 with activation of NF-*κ*B and subsequent production of proinflammatory cytokines such as IL-6 and IL-8 [24, 27, 28, 34, 35]. Microbiota dysbiosis is associated with reduction of short-chain fatty acids, anti-inflammatory molecules that regulate the immune response in the intestine; these fatty acids ameliorate the intestinal inflammation in IBD in an animal model [36]. However, it is possible that downregulation of ABCC7/CFTR contributes to IL-17 upregulation in patients with UC allowing the persistent activity as we observed in the active group in our study [30, 31]. A previous study demonstrated an upregulation of the expression of the CFTR mRNA of fivefold in mildly active UC, 2.4-fold in moderate, and 1.5-fold in severely active UC compared with normal control. Our results differ from this study, because our active UC group consisted mostly of moderate to severe inflammation where they found lower expression of CFTR in moderate to severe compared to mild active inflammation and controls [37].

## 5. Conclusions

A downregulation of gene and protein expression of CFTR was found in patients with active UC suggesting the role of CFTR in the development of colonic inflammation. The CFTR downregulation was associated with the clinical course of UC.

## Figures and Tables

**Table 1:** Clinical characteristics of UC patients and controls.

Clinical characteristics	Active UC *N* = 20		Remission UC *N* = 21		Controls *N* = 21	
	*n*	%	*N*	%	*n*	%
Gender						
Male	10	50	10	47.6	10	47.6
Female	10	50	11	52.4	11	52.4
Age (median, range)	40	(24–72)	47	(16–75)	44	(25–74)
Age at diagnosis (mean, SD)	31	(6–56)	34	(9–66)		
Years of evolution (median, range)	6	(1–24)	7	(0–17)		
Extent of disease						
E1	6	30	6	28		
E2	7	35	7	34		
E3	7	35	8	38		
Activity of UC						
Mi	2	10				
Mo	9	45				
Se	9	45				
Extraintestinal manifestation						
Present	10	50	11	52.3		
Absent	10	50	10	47.7		
Clinical course of disease						
Initially active	7	35	7	33.3		
Intermittent	7	35	7	33.3		
Continuous	6	30	7	33.3		
Medical treatment						
5-ASA	20	100	21	100		
Steroids	7	35	0	0		
Thiopurines	4	20	5	23.8		

**Table 2:** Association between gene expression of ABCC7/CFTR and clinical characteristics of UC patients.

Clinical characteristics	*n*	%	ABCC7/CFTR expression	*P* value
Gender				
Male	21	52		
Female	20	48		
Age at diagnosis				
<40	34	83	19.37	0.745
>40	7	17	28.93	
Extraintestinal manifestations				
Present	27	65	19.24	0.191
Absent	14	35	24.39	
Extent of disease				
Distal colitis	19	46	21.71	0.724
Pancolitis	22	54	20.39	
Years of evolution				
<3	7	17	14.57	0.119
>3	34	83	22.32	
Clinical course of disease				
Long term remission	31	76	25.64	**0.005** ^∗^
Intermitent activity	5	12	20.78	
Persistent activity	5	12	14.63	

^∗^OR = 21.7, 95% CI: 3.59–132.0.

## Data Availability

The data availability will be provided upon request.
